# Laser Hemorrhoidoplasty Versus Ligasure Hemorrhoidectomy: A Comparative Analysis

**DOI:** 10.7759/cureus.43119

**Published:** 2023-08-08

**Authors:** Cemalettin Durgun, Ebral Yiğit

**Affiliations:** 1 General Surgery, Üsküdar University Faculty of Medicine, Istanbul, TUR; 2 General Surgery, Memorial Dicle Hospital, Diyarbakır, TUR; 3 General Surgery, Gazi Yasargil Training and Research Hospital, Diyarbakir, TUR

**Keywords:** hemorrhoidal disease, ligasure, hemorrhoidectomy, lhp, laser hemorrhoidoplasty

## Abstract

Background and objective

Minimally invasive techniques in the surgical treatment of hemorrhoids have been gaining in popularity. Laser hemorrhoidoplasty (LHP) and LigaSure™ (LigH; Medtronic, Dublin, Ireland) hemorrhoidectomy methods are the most recent innovative methods that are increasingly used. In this study, we aimed to compare the effectiveness of these two innovative techniques.

Methods

The data of patients who underwent LHP or LigH for grade III hemorrhoidal disease at our clinic between January 2022 and June 2023 were retrospectively analyzed. Postoperative pain levels, time to return to work or daily activities, complication rates, and recurrence rates of the treated patients were recorded.

Results

A total of 100 patients were included in the study. Of these, 48 patients had LHP surgery and 52 had LigH surgery. The demographic characteristics of both groups were similar. The mean operation time was statistically significantly shorter in the LHP group (p<0.001). The visual analog scale (VAS) scores on postoperative days one and seven were lower in favor of the LHP (2.4 ± 0.7 and 1.2 ± 0.9 vs. 6.2 ± 1.5 and 3.8 ± 1.3, respectively; p< 0.001). The median time to return to daily activity was 2.3 (range: one to three) days in the LHP group and 4.6 (range: 3-11) days in the LigH group (p<0.001). Recurrence was observed in 11 (22%) patients in the LHP group and in three (6%) patients in the LigH group (p<0.001).

Conclusion

Based on our findings, LHP is an effective procedure for the surgical treatment of hemorrhoidal disease as it is associated with less morbidity, less pain, early return to work, and acceptable recurrence rates.

## Introduction

Hemorrhoids are defined as the enlargement and distal displacement of the normal anal cushions that are made of loose connective tissue, smooth muscle, and arterial and venous vessels [1]. Its prevalence ranges from 38.9% to 44.7% in colonoscopy screening [2]. Among these, symptomatic hemorrhoids account for 4.4% [3]. Prolapse, wetness, swelling, pain, bright red rectal bleeding, itching, and burning are the most typical signs of hemorrhoids.

Low-grade hemorrhoids can be treated with medical intervention, dietary supplements, and lifestyle changes without the need for surgery. However, symptomatic high-grade hemorrhoids that have failed conservative medical therapy require surgery. Many methods have been described to date for the surgical treatment of hemorrhoids. Milligan-Morgan hemorrhoidectomy and Ferguson hemorrhoidectomy are the gold-standard traditional surgical methods; however, these methods are associated with severe postoperative pain as well as a risk of bleeding and anal stenosis.

LigaSure™ (LigH; Medtronic, Dublin, Ireland) is a vessel sealing system that completely coagulates blood vessels up to 7 mm in diameter with minimal thermal damage to the surroundings. It has been shown to be effective in reducing anal spasms and pain after hemorrhoidectomy [4]. In addition, It has been associated with significantly less surgical time and bleeding compared to traditional hemorrhoidectomy [5].

Recently, non-excisional procedures have been gaining popularity in the surgical treatment of symptomatic hemorrhoids. Laser hemorrhoidoplasty (LHP) is the most current among these technologies. LHP is a method in which hemorrhoidal tissue is coagulated with a laser probe by entering through the base of the hemorrhoids. Low postoperative pain and satisfactory symptom relief at long-term follow-ups have been reported with laser therapy [6].

In this study, we aimed to compare the efficacy of the two most recent techniques, LHP and LigH, in the surgical treatment of hemorrhoidal disease.

## Materials and methods

This retrospective study involved 100 patients who underwent surgery for hemorrhoids at Memorial Dicle Hospital between January 2022 and June 2023. Patients were classified into two groups according to the type of procedure: patients who underwent LigH and those who underwent LHP. The inclusion criteria were as follows: adult patients older than 18 years of age with grade III hemorrhoids that did not respond to medical treatment. The exclusion criteria were as follows: patients with inflammatory bowel disease, patients with a history of previous hemorrhoid surgery, patients with bleeding disorders, and those with acute thrombosed hemorrhoids.

All patients included in the study had received medical treatment at least twice in the six months preceding the operation and had not benefited sufficiently from it. Before surgery, colonoscopy had been performed in patients over 50 years of age while the rest had undergone rectosigmoidoscopy. The patients who had undergone surgery were invited for follow-ups at one week and one month postoperatively. All patients were contacted by phone before the study and invited for the last follow-up.

The current study was approved by the Memorial Bahçelievler Hospital Ethics Committee (approval no: 08.06.2023/99). The research was conducted according to the tenets of the Declaration of Helsinki. Written informed consent was obtained from each patient included in the study.

Surgical technique

All patients in both groups were operated under spinal anesthesia in the lithotomy position. Antibiotic prophylaxis with 1 g of third-generation cephalosporin IV was administered to all patients. Bowel cleansing was performed by rectal enema half an hour before surgery. In the postoperative period, oral ciprofloxacin 500 mg and oral dexketoprofen 25 mg were given twice daily.

In the LigH group, hemorrhoidectomy was performed with LigaSure^TM^ Small Jaw® (Medtronic, Dublin, Ireland). The anodermal wedge was incised with an electrocautery or scalpel. After this level, en-bloc excision of hemorrhoidal pockets was performed with LigaSure up to the anorectal ring. A compressive hemostatic sponge was placed in the anal canal and the operation was terminated.

In the LHP group, a 2-mm skin incision was made lateral to the anal verge at the base of each hemorrhoidal pocket. The laser probe was advanced through the submucosal tissue to the proximal end of the hemorrhoidal pocket. Then, using a 1470 nm diode laser generator (LEONARDO® DUAL 45 Biolitec®, Jena, Germany), three pulses of 8 watts, of approximately 24 Joules, were delivered in three seconds each. Two more pulses were delivered in the submucosal area while the probe was retracted. An ice finger made of a glove was placed in the anal canal, and after one minute, an anal sponge was then inserted into the anal canal and the operation was completed.

Mean operative times were recorded for all patients. Postoperative pain was assessed by visual analog scale (VAS) on the first day and at the first week. Postoperative bleeding was evaluated at one-day and one-week follow-ups and classified as spontaneous, post-defecation, or no bleeding. Time to return to daily activity was assessed in all patients.

Statistical analyses were performed with IBM SPSS Statistics for Windows, Version 23.0. (IBM Corp., Armonk, NY). Categorical descriptive data were presented as numbers and percentages, and continuous descriptive data were expressed as mean ± standard deviation (SD). Results summarized as means were analyzed by unpaired t-test, results summarized as median were analyzed by the Mann-Whitney U test, and results summarized as percentages were analyzed by Fisher's exact test. A p-value <0.05 was considered statistically significant.

## Results

A total of 100 patients who underwent LHP or LigH for grade III hemorrhoidal disease at our clinic from January 2022 to June 2023 and met the inclusion criteria were included in the study. Fifty-two patients underwent the LigH procedure and 48 underwent the LHP procedure. The mean age was 39 ± 11 (range: 24-70) years in the LigH group and 37 ± 8 (range: 22-56) years in the LHP group (p=0.128). As for gender, 17 (35.4%) patients in the LHP group and 22 (42.3%) patients in the LigH group were female (p=0.209). The median hospital stay was 1.14 ±0.35 (one to two) days in the LigH group and 0.80 ±0.40 (zero to one) days in the LHP group (p=0.002). The mean operative time was 16.05 ± 3.6 (10-22) minutes and 23.13 ± 4.15 (14-32) minutes in the LHP and LigH groups, respectively (p=0.0002).

No intraoperative complications were observed in either group, and no bleeding requiring surgical intervention occurred in the postoperative period. Bleeding after defecation occurred on the first postoperative day in 12 (24%) patients in the LHP group. No bleeding was observed on the seventh day after the operation. In the LigH group, 32 (64%) patients reported bleeding in the form of a few drops after defecation on postoperative day one. In the follow-up performed on the seventh postoperative day, it was reported that 18 (36%) patients had continued to experience bleeding in the form of a few drops during defecation, but the amount had decreased (p<0.0001). The number of operated hemorrhoid pockets was 2.71 and 2.76 in LigH and LHP groups, respectively. There was no statistically significant difference between the groups (p=0.381).

The mean postoperative pain score assessed by the VAS was significantly lower in the LHP group. On postoperative day one, the VAS score was 2.4 ± 0.7 in the LHP group and 6.2 ± 1.5 in the LigH group (p<0.001). On postoperative week one, the VAS score was 1.2 ± 0.9 in the LHP group and 3.8 ± 1.3 in the LigH group (p<0.001). The median time to return to daily activity was 2.3 (range: one to three) days in the LHP group and 4.6 (range: 3-11) days in the LH group (p<0.001). Thrombosis of the external hemorrhoids was observed in one patient in the LHP group a week after the procedure. The patient recovered with medical treatment. No thrombosed hemorrhoids were observed in the LigH group. Wound infection was not observed in any patient. Anal stenosis was not seen in either group of patients during the entire follow-up period. In our study, the mean follow-up time was 12 ± 3 (7-18) months.

The percentage of patients with recurrent hemorrhoids was statistically significantly higher in the LHP group than in the LigH group. It was reported in 11 (22%) patients in the LHP group and in three (6%) patients in the LigH group (p<0.001). Demographic and operative characteristics are detailed in Table 1.

**Table 1:** Demographic and operative characteristics *Unpaired t-test; **Fisher’s exact test; ***Mann-Whitney U test p< 0.05 was considered statistically significant LHP: laser hemorrhoidoplasty; LigH: LigaSure hemorrhoidectomy; SD: standard deviation; POD: postoperative day; VAS: visual analog scale

Variables	LHP	LigH	P-value
Age, years, mean ± SD (range)	39 ± 11 (24–70)	37 ± 8 (22–56)	0.128*
Gender, male/female, n (%)	17 (35.4%)/31 (64.6%)	22 (42.3%)/30 (57.7%)	0.209**
Operative time, minutes, mean ± SD (range)	16.05 ± 3.6 (10–22)	23.13 ± 4.15 (14–32)	<0.001*
Hospital stay, days, mean ± SD (range)	0.80 ± 0.40 (0–1)	1.14 ± 0.35 (1–2)	0.00245*
VAS score at 1st POD. mean ± SD	2.4 ± 0.7	6.2 ± 1.5	< 0.001*
VAS score at 1st postoperative week, mean ± SD	1.2 ± 0.9	3.8 ± 1.3	<0.001*
Time to return to daily activities, days, median (range)	2.3 (1–3)	4.6 (3–11)	<0.001***
Recurrence, n (%)	11 (22%)	3 (6%)	<0.001

## Discussion

Many methods have been reported to be used successfully in the treatment of hemorrhoids, but there is still no established "gold standard" modality [7]. LHP and LigH are recently designed techniques that help reduce complications of the surgical procedure and have been compared to other conventional hemorrhoidectomy procedures in many published studies. Both methods have been reported to improve postoperative patient satisfaction.

The LigaSure device is a vessel sealing system that can coagulate vessels up to 7 mm in diameter and does not require sutures [8]. In LigH, thermal spread to surrounding tissues is minimal, resulting in less tissue damage. Compared to traditional hemorrhoidectomies, LigH has less postoperative pain and less need for postoperative analgesic use [9]. Furthermore, it results in less intraoperative bleeding and shorter operation time [8].

LHP is a novel minimally invasive procedure used for the surgical treatment of symptomatic hemorrhoids. Laser therapy induces shrinkage and degeneration of hemorrhoidal tissue, leading to submucosal protein denaturation and cellular fibrosis. This fibrotic process results in adhesion to the underlying tissue, effectively preventing the prolapse of the hemorrhoidal pocket [10,11]. This results in hemorrhoidal tissue volume reduction, similar to that in conventional hemorrhoidectomy, without the need for physical tissue excision [12]. LHP has been reported to have advantages over hemorrhoidectomy both intraoperatively and postoperatively [12]. It has been reported that patients who undergo LHP have less postoperative pain and morbidity. Reduction in postoperative pain leads to a decrease in drug complications resulting from analgesic use and increased patient satisfaction [13]. Patients after LHP have less postoperative pain and can return to work or daily activities earlier [13]. This may be explained by the fact that in LHP, tissue excision is not performed below the dentate line where pain fibers are dense [14]. In our study, postoperative pain and the need for analgesic use were significantly lower in the LHP group. Patients in the LHP group had significantly lower VAS scores on postoperative days one and seven than those in the LigH group.

The main advantage of LHP is a faster return to work and normal life. Several studies have reported that all patients return to their normal daily activities within two days after LHP [15]. In our study, the mean time to return to daily activities after LHP was significantly lower [2.3 (range: one to three) days] compared to that after LigH [4.6 (range: 3-11) days]. LHP has a significantly shorter operative time and less intraoperative blood loss compared to conventional hemorrhoidectomy. In a meta-analysis, the mean operation time for LHP was reported to be 12 minutes, while intraoperative blood loss was reported to be 19 ml on average [12]. In our study, the mean operation time was 16.05 ± 3.6 minutes in the LHP group and 23.13 ± 4.15 minutes in the LigH group. Intraoperative bleeding was negligible in both groups.

Although intraoperative blood loss is insignificant in both LHP and LigH methods, a small amount of bleeding during defecation in the postoperative period persists longer in the LigH group. In a meta-analysis comparing laser hemorrhoidoplasty with conventional hemorrhoidectomy, Wee et al. reported that the risks of bleeding (p>0.999), prolapse (p=0.240), and complete resolution (p=0.240) were not statistically significant at the 12-month follow-up [12].

Varying recurrence rates have been described in the literature at different follow-up periods after LHP. Weyand et al. have reported a recurrence rate of 8.8% in a six-month follow-up period [16], while Faes et al. have reported a recurrence rate of 34% in a five-year follow-up period [17]. It is clear that the recurrence rate increases as the follow-up period increases. High recurrence rates weaken the success rate of this newly developed technique. In our study, the recurrence rate during the follow-up period was higher in the LHP group compared to the LigH group: 11 (22%) vs. three (6%).

LHP also offers the advantage of low postoperative and intraoperative complication rates. In our study, although no significant complications were observed in either group, LHP was superior in terms of postoperative pain, bleeding, and return-to-work time, and therefore LHP seems to be superior in terms of patient satisfaction in the early postoperative period.

This study has a few limitations, such as the retrospective nature of the study, the relatively short follow-up time, and the small sample size.

## Conclusions

LHP has been shown to provide favorable clinical outcomes in the short term as a non-excisional procedure. It offers benefits such as reduced morbidity and pain, as well as earlier return to work or daily activities. Additionally, LHP appears to have acceptable recurrence rates in the medium term. Although the results of studies on these new surgical techniques with fewer complications are encouraging, more prospective studies with larger sample sizes and longer follow-up periods are needed.
